# Data in support of dyslipidemia-associated alterations in B cell subpopulations frequency and phenotype during experimental atherosclerosis

**DOI:** 10.1016/j.dib.2016.02.048

**Published:** 2016-03-05

**Authors:** Héctor Rincón-Arévalo, Diana Castaño, Janny Villa-Pulgarín, Mauricio Rojas, Gloria Vásquez, Luis A. Correa, José R. Ramírez-Pineda, Lina M. Yassin

**Affiliations:** a Grupo de Inmunología Celular e Inmunogenética, Instituto de Investigaciones Médicas, Facultad de Medicina, Universidad de Antioquia UdeA, Calle 70-52-21, Medellín, Colombia; b Grupo Inmunomodulación, Instituto de Investigaciones Médicas, Facultad de Medicina, Universidad de Antioquia UdeA, Calle 70-52-21, Medellín, Colombia; c Unidad de Citometría, Facultad de Medicina, Sede de Investigación Universitaria, Universidad de Antioquia UdeA, Calle 70-52-21, Medellín, Colombia; d Sección de Dermatología, Departamento de Medicina Interna, Facultad de Medicina, Universidad de Antioquia. Laboratorio Clínico VID, Obra de la Congregación Mariana, Medellín, Colombia; e Grupo de Ciencias Básicas, Universidad CES, Medellín, Colombia

**Keywords:** *apoE*^*−/−*^, apolipoprotein-E-deficient, FBS, Fetal Bovine Serum, HFD, high fat diet, MFI, Mean fluorescence intensity, PBS, phosphate buffered saline, SLD, standard lab diet, B cells, Atherosclerosis, Apolipoprotein E, Cholesterol, Dyslipidemia

## Abstract

Cardiovascular diseases are the most common cause of death in the world, atherosclerosis being its main underlying disease. Information about the role of B cells during atherosclerotic process is scarce, but both proatherogenic and atheroprotective properties have been described in the immunopathology of this disease. Frequency and phenotype of B cell subpopulations were studied in wild type and apolipoprotein-E-deficient (*apoE*^*−/−*^) mice fed or not with high-fat diet (HFD), by flow cytometry. Here, we provide the information about the materials, methods, analysis and additional information related to our study published in Atherosclerosis (DOI: 10.1016/j.atherosclerosis.2015.12.022, article reference: ATH14410) [1]. The data contained in this article shows and supports that mice with advanced atherosclerosis have a variety of alterations in frequency and phenotype of B cell subsets, most of which associated with dyslipidemia.

**Specifications Table**

**Table:** 

Subject area	Immunology.
More specific subject area	Immunology of Atherosclerosis.
Type of data	Figures and materials and methods.
How data was acquired	Flow cytometry, microscopy, ELISA and conventional wet chemistry.
Data format	Raw and analyzed.
Experimental factors	Type of mouse: Wild type and *apoE*^−^^*/−*^.
Diet: Standard laboratory diet (SLD) and high fat diet (HFD).
Experimental features	Design that encompasses both *in vivo* and *in vitro* observations of B cell subsets in atherosclerosis established and healthy control mice.
Data source location	Medellin, Colombia.
Data accessibility	Data is within this article.

**Value of the data**

• Similar alterations in frequency and phenotype of B cell subsets could be presented in murine models of other chronic inflammatory diseases.
• The data can be used for further investigations concerning B cells biology in other lipid associated and metabolic diseases.
• The data and procedures can be used by other scientists investigating the effects of dyslipidemia on murine B cells.
• Characterization strategy for B cell subsets presented herein, can be used in other murine models.

## 1 Data

Here we describe detailed materials and methods, the analysis strategy for discriminating B cells from aortas, lymph nodes and spleens and some aspects of the phenotype of B cell subsets in the atherosclerosis mouse model *apoE*^*-/-*^ and control mice (Fig. 1, Fig. 2, Fig. 3, Fig. 4, Fig. 5, Fig. 6, Fig. 7, Fig. 8, Fig. 9, Fig. 10, Fig. 11, Fig. 12, Fig. 13, Fig. 14). A similar approach for the analysis of B cells in *apoE^-/-^* mice were previously published in [1].

## 2 Experimental design, materials and methods

### 2.1 Mice

C57BL/6 WT and *apoE*^*-/-*^ mice were purchased from Charles River (MI) and Jackson Laboratory (ME), respectively. Mice were housed and kept under a specific pathogen-free environment, at the animal facility, Sede de Investigación Universitaria, Universidad de Antioquia (Medellín, Colombia). The institutional animal ethical committee at Universidad de Antioquia approved all the animal protocols and experiments. Water and a standard laboratory diet (SLD, Labdiet Company, IN) or high fat diet (HFD; 1.5% cholesterol, 42% anhydrous milk fat) (Harlan Teklad, WI) were given *ad libitum* to the animals.

### 2.2 Sample collection

Mice were euthanatized with CO_2_ exposition and heart, spleen, lymph nodes and aorta were harvested by dissection. Hearts were embedded in paraformaldehyde solution (4%, JT Baker, NJ) at 4 °C during 48 h, and then in sucrose solution (30%, Sigma-Aldrich, MO) for 24 h. Finally, heart tissues were stored at −20 °C in Shandon cryomatrix (Thermo Scientific, PE) until their use. Spleens, aortas and lymph nodes were collected in phosphate buffered saline (PBS, Gibco, NY) at 4 °C and immediately processed. Peripheral blood obtained by cardiac puncture was centrifuged at 10.000×*g* during 10 min at 4 °C to obtain serum. After centrifugation, serum was stored at −20 °C until used.

Heart tissues were serially sectioned in a CM-1850 Cryostat (Leica Microsystems, Germany) for 6–7 µm thickness on slides with positive charge (Thermo scientific), and stained with Hematoxylin–Eosin (Sigma-Aldrich). Microphotografies were taken from aortic root and ascendant aorta at 4× with a digital camera Nikon DS-Fi1 coupled to a Nikon E2000 microscope (Nikon, Japan). Images were analyzed with NIS Element Software (Nikon) to determinate the size of atherosclerotic lesions.

### 2.3 Antibodies and other reagents for flow cytometry

The following monoclonal antibodies were purchased from Becton Dickinson Bioscience (BD Bioscience, CA): CD3e-FITC (145-2C11), CD4-FITC (RM4-5), CD5-PECy5 (53-7.3), CD8a-PE (53-6.7), CD11b-APC (M1/70), CD11c-APC (HL3), CD19-PECy7 (1D3), CD21-FITC (7G6), CD23-PE (B3B4), CD24-APC (M1/69), CD40-APC (3/23), CD45-APCCy7 (30-F11), CD49b/Pan-NK-FITC (DX5), CD80-APC (16-10A1), CD86-APC (GL1), CD95-PE (15A7), IgM-biotin (II/41), IgD-V450 (11-26c.2a), Ter-119-PE (TER-119), B220-Pacifc Blue (RA3-6B2), and the respective isotype controls. LIVE/DEAD Fixable Aqua Dead Cell Stain (Invitrogen, CA) was performed for viability study in each sample. Streptavidin PE (One Lambda, CA) and V450 (BD Bioscience) were used for biotin labeled antibodies.

### 2.4 Isolation of murine splenocytes

Spleens were perfused and macerated with syringe plunger (Rymco, Colombia) and cold media compound of RPMI 1640 Glutamax (Gibco) with 2% inactivated Fetal Bovine Serum (FBS, Gibco) and 1% Penicillin/Streptomycin (Gibco) on ice. Cells were pelleted at 800×*g*, for 10 min at 4 °C. Erythrocytes were lysed with RBC Lysis Buffer (eBiosciences, CA) for 5 min with constant movement. Splenocytes were washed with PBS, resuspended in cold media and immediately used after obtention. Number of cells was determined in Neubauer camera. Viability (around 90%, **data not shown**) was evaluated by trypan blue exclusion and Live/Dead Fixable Aqua Dead Cell Stain Kit (Invitrogen).

### 2.5 Isolation of aortic cells

Aortas were processed as previously reported [2]. Each aorta was washed in cold PBS before the treatment and were cut with sterilized scissors in 1 mL of enzymatic cocktail, containing 450 U/ml Collagenase I (Sigma-Aldrich), 125 U/ml Collagenase XI (Sigma-Aldrich), 60 U/ml DNAse I (Sigma-Aldrich) and 60 U/ml Hyaluronidase I (Sigma-Aldrich) for 1 h at 37 °C. Cell suspensions were passed through a 74 µm mesh, centrifuged at 400×*g* for 5 min at 4 °C and suspended in cold media. Cells were processed immediately after obtention and their number and viability was determined with trypan blue exclusion and Live/Dead Fixable Aqua Dead Cell Stain Kit (>75%, **data not shown**). Blood contamination was determined with TER-119 (<18%, **data not shown**).

### 2.6 Multiparametric flow cytometry

Cell suspensions (1×10^6^ splenocytes and 1–2×10^5^ aortic cells) were washed in 1 ml of FACS buffer (0.01% sodium azide (Sigma-Aldrich) and 3% FBS in PBS, pH=7.35) and centrifuged at 600×*g* for 5 min, 4 °C. Cell pellets were incubated with blocking buffer (10% FBS, 0.1% bovine serum albumin (Sigma-Aldrich) and 0.01% sodium azide in PBS, pH=7.35) for 15 min at 4 °C. Specific monoclonal antibodies were added in 100 µl FACS buffer and incubated for 20 min at 4 °C in dark. Cells marked with biotinylated antibodies were washed twice with FACS buffer and incubated with streptavidin for another 20 min at 4 °C in dark. Cells were washed two times with FACS buffer and immediately acquired in the flow cytometer FACS Canto-II (BD Biosciences). Data were analyzed using FlowJo software (Tree star, CA) and reported as percentages, absolute numbers and median fluorescence intensity (MFI).

### 2.7 Immunochemistry

Paraffin-embedded sections of spleen, lymph nodes and aorta were cut (4 μm) and stained with Hematoxylin-Eosine, anti- CD3, CD20, CD23, CD68 and CD79a. Images were acquired using the 4X, 10X, 20X objective on a Leica DM500 coupled with digital camera (Leica, Germany) using Leica LAS EZ software (Leica).

### 2.8 Determination of cholesterol levels

Total cholesterol serum levels were determined by conventional wet chemistry method using an A-15 automated biochemical analyzer (Biosystems, Spain) in the School of Veterinary, Universidad de Antioquia.

### 2.9 Immunoglobulin levels

Serum IgM and IgG1 levels were measured by Enzyme-linked Immunosorbent Assay (ELISA) Ready-Set-Go! Kits (eBiosciences), following manufacturer׳s instructions and using an ELx800 Absorbance Microplate Reader (Biotek instruments, VT) at 450 nm emission.

### 2.10 Cell culture

Culture media containing 10 mM L-glutamine (Gibco), 0.05 mM 2-mercaptoethanol (Gibco), 1% Penicillin/Streptomycin in RPMI 1640 Glutamax. Two types of serum were used: 20% lipemic serum from *apoE*^*−/−*^HFD mice 20 weeks old, and 20% non-lipemic serum from WT-SLD mice 20 weeks old. Total splenocytes (2×10^6^) were cultured in 1 ml of media for 5 and 12 hours at 37 °C and 5% CO_2_. Cells were washed with PBS and the immunofluoresce with specific antibodies was performed as previously described. Cells were fixed with a commercial solution (fixation/permeabilization kit, Foxp3/Transcription Factor, eBioscience) during 1 h and washed twice with PBS. Finally, cells were stained with Nile Red (Sigma-Aldrich) for 15 min at 4 °C in dark. Cells were washed twice with PBS and immediately acquired in the flow cytometer.

### 2.11 Statistical analysis

Differences between groups were examined using two ways ANOVA with Šidák´s multiple comparisons post-test. Differences in MFI of CD95 and Nile Red between groups were examined using non-parametric Mann–Whitney test. Spearman rank correlation coefficient test was performed to assess the association between two variables. Data was analyzed in Prism 6.0 software (GraphPad, CA). *P*-values <0.05 were considered statistically significant. The classical principal component analysis was performed with the Statgraphics Centurion XVI software (StatPoint Technologies, VA).

## Figures and Tables

**Fig. 1:**
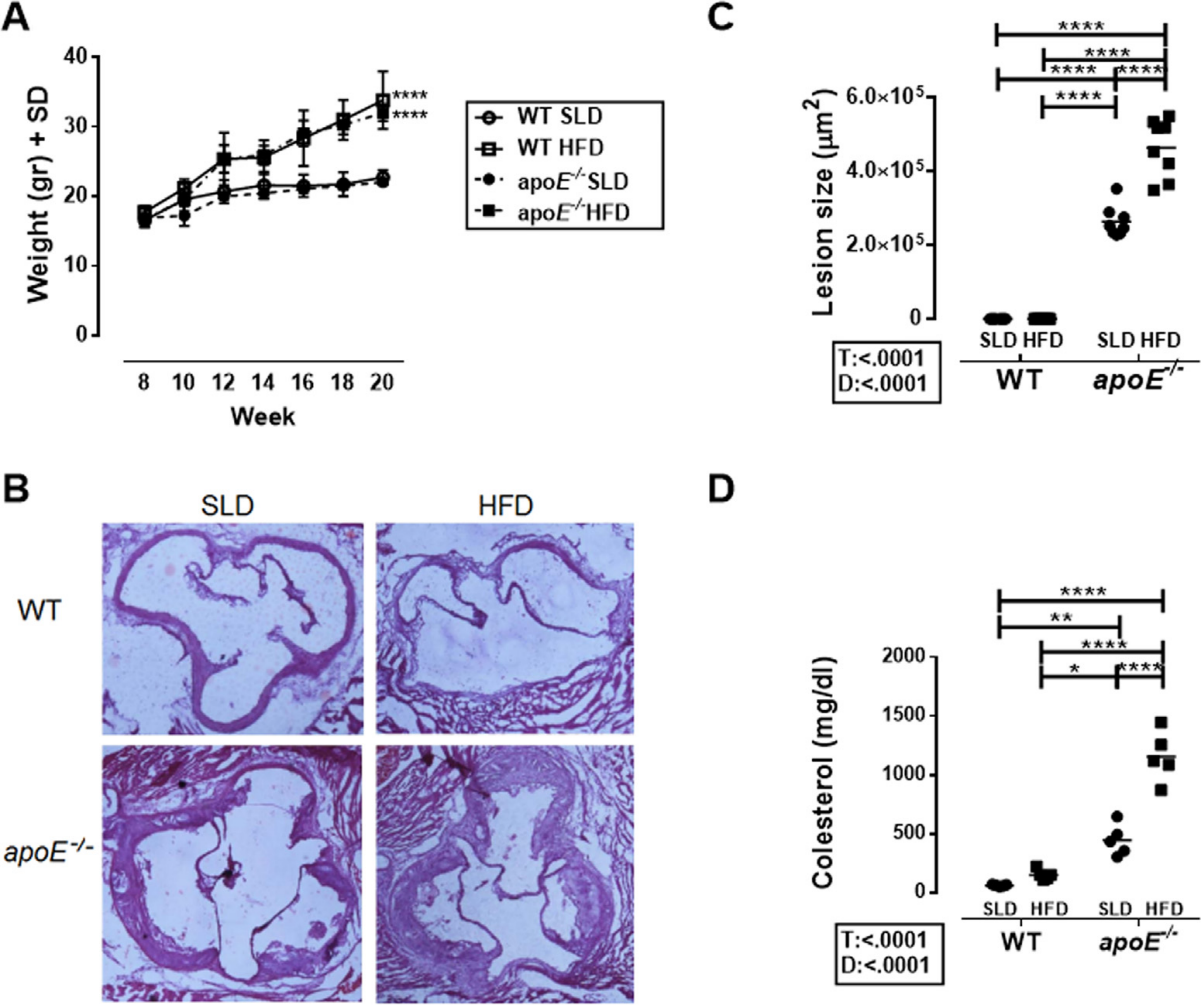
**Mouse model of atherosclerosis. A.** Weight of mice from the four study groups from week 8 until week 20. The data are shown as mean±standard deviation (SD) of 7 mice per group are shown. **B.** Representative photomicrograph of aortic sinus cuts stained with Hematoxylin-Eosin for each of the study groups. **C.** Mean atherosclerotic plaque size (μm^2^) from 8 mice per group. **D.** Serum cholesterol levels in the study groups. Mean and data from 5 mice per group. Two-way ANOVA with Šídák post-test. The sources of variation (diet (D) and type of mice (T)) are presented in the boxes under the Y-axis of each graphic, only where significant differences where observed. **** p<0.0001, *** p<0.001, ** p<0.01 and * p<0.05.

**Fig. 2:**
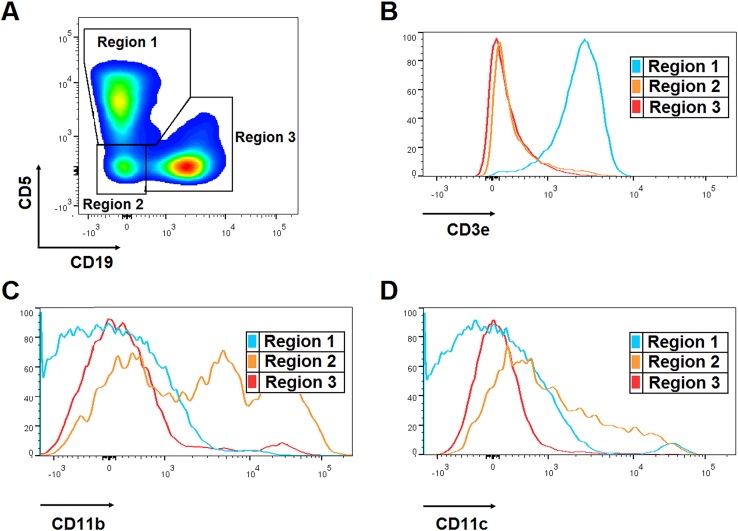
**Analysis of splenic B cells. A.** Representative density plot of splenocytes from a WT-SLD mouse that shows the selection of B cells according to CD5 and CD19 expression, which allowed the determination of three populations: CD19^-^CD5^+^ (region 1), CD19^-^CD5^-^ (region 2) and CD19^+^CD5^+/-^ (region 3, B cells). Representative histograms of the expression of **B.** CD3, **C.** CD11b and **D.** CD11c on splenocytes from a WT-SLD mouse in the regions indicated in **A**.

**Fig. 3:**
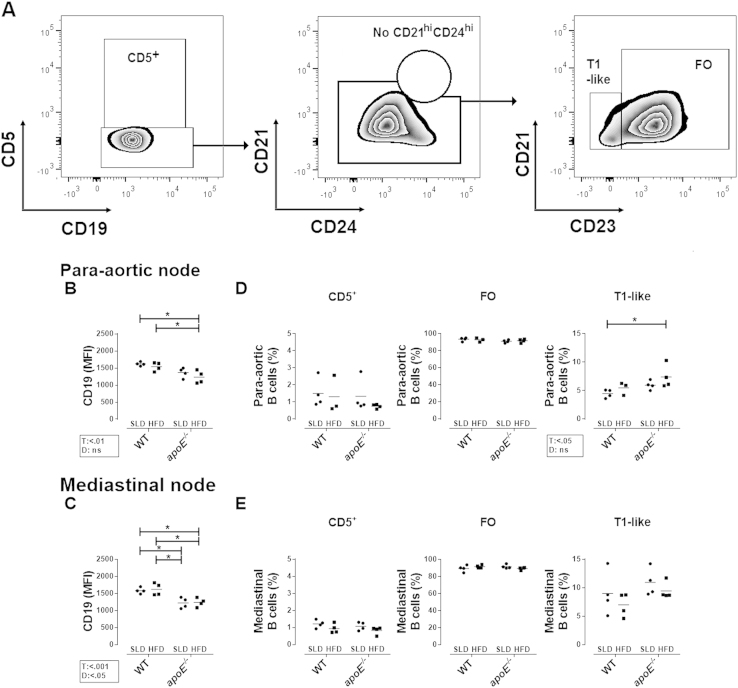
**B cells from para-aortic and mediastinal lymph nodes in mice with atherosclerosis. A.** Representative analysis of B cell subpopulations from lymph nodes divided in CD5^+^, T1-like and FO, from WT-SLD mouse by flow cytometry. Mean fluorescence intensity (MFI) of CD19 on B cells from **B.** para-aortic and **C.** mediastinal lymph nodes. Frequency of B cell subpopulations in **D.** para-aortic and **E.** mediastinal lymph nodes. Mean and data from 3-4 mice per group are shown. Analysis in lymph nodes was performed as in spleens. Two-way ANOVA with Šídák post-test were performed.

**Fig. 4:**
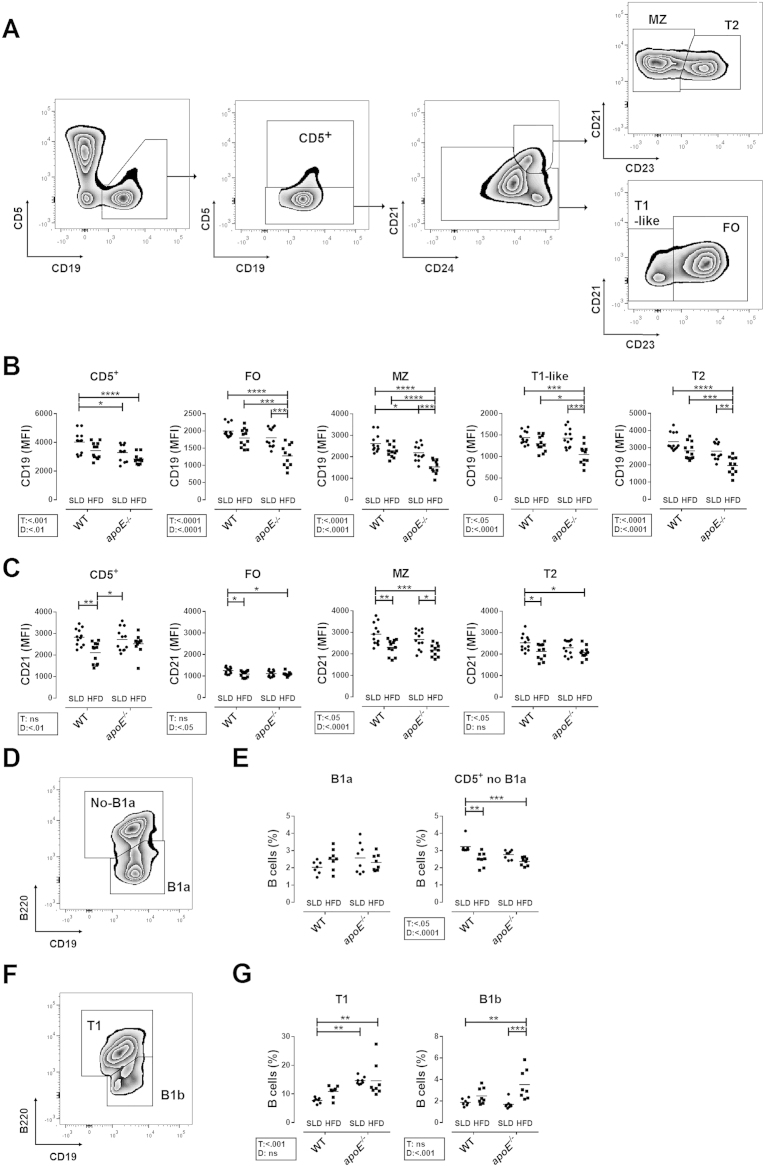
**Analysis of splenic B cell subpopulations. A.** Representative analysis of splenic B cell subpopulations from a WT-SLD mouse. According to the expression of CD5, CD19, CD21, CD23 and CD24, lymphocytes were divided into CD5^+^, MZ, T1-like, T2 and FO subsets. MFI of **B.** CD19 and **C.** CD21 on B cell subpopulations. MFI from 11-12 mice per group are shown from three independent experiments. **D.** Representative density plot of B220 expression on CD5^+^ B cells of a WT-SLD mouse. **E.** Frequency of B1a and no-B1a subpopulations in splenic B cells. **F.** Representative density plot of B220 expression on T1-like cells of a WT-SLD mouse. **G.** Frequency of B1b and T1 subpopulations in splenic B cells. Mean and data from 7-8 mice per group are shown. Two-way ANOVA with Šídák post-test were performed.

**Fig. 5:**
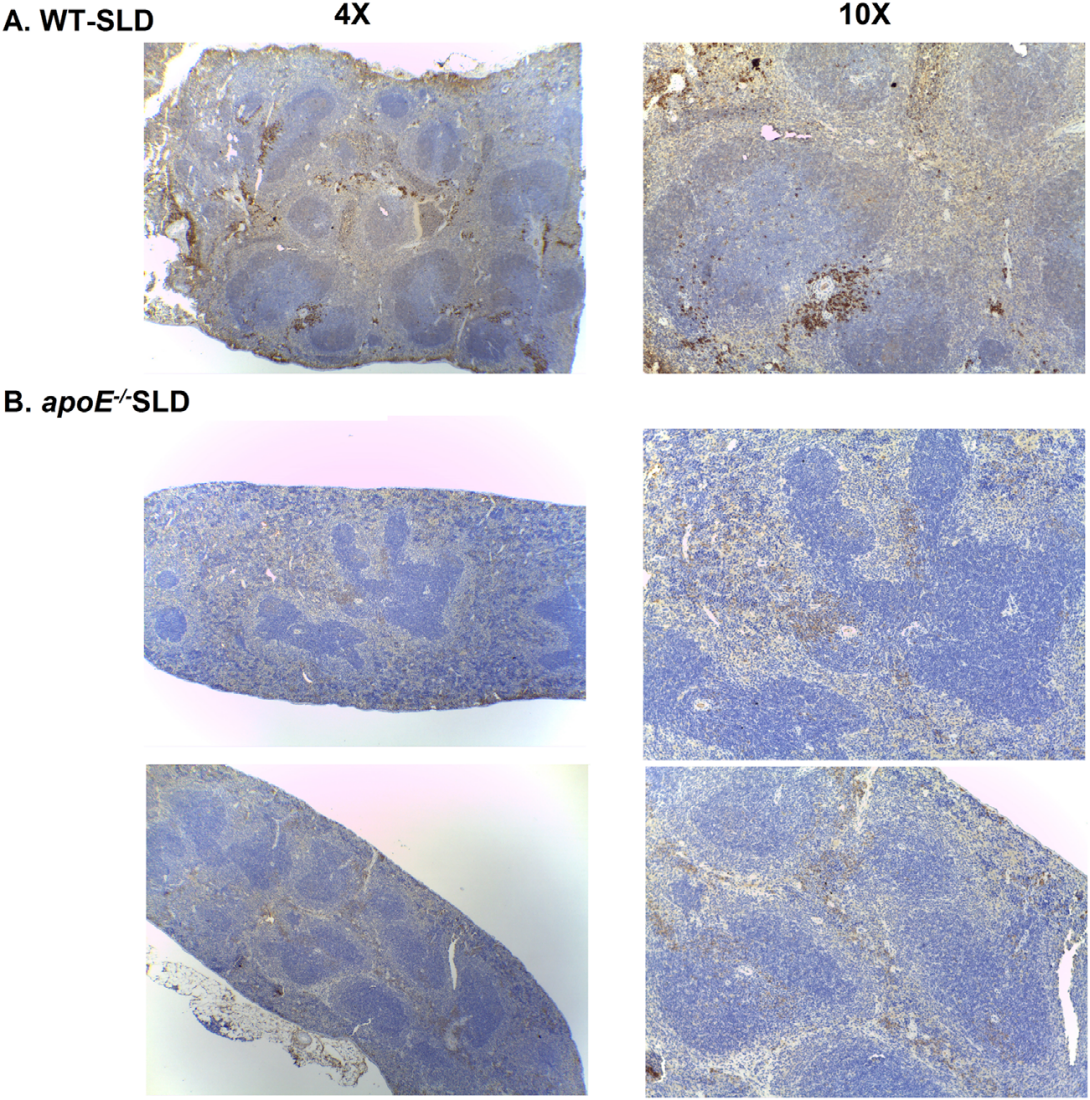
**Decreased CD20 labeling of splenic cells from*****apoE***^***-/-***^**SLD.** Representative photographs of CD20 staining (brown) at 4X (left) and 10X (right) magnification of paraffin-embedded sections of spleens from **A.** WT-SLD and **B.***apoE*^*-/-*^SLD mice.

**Fig. 6:**
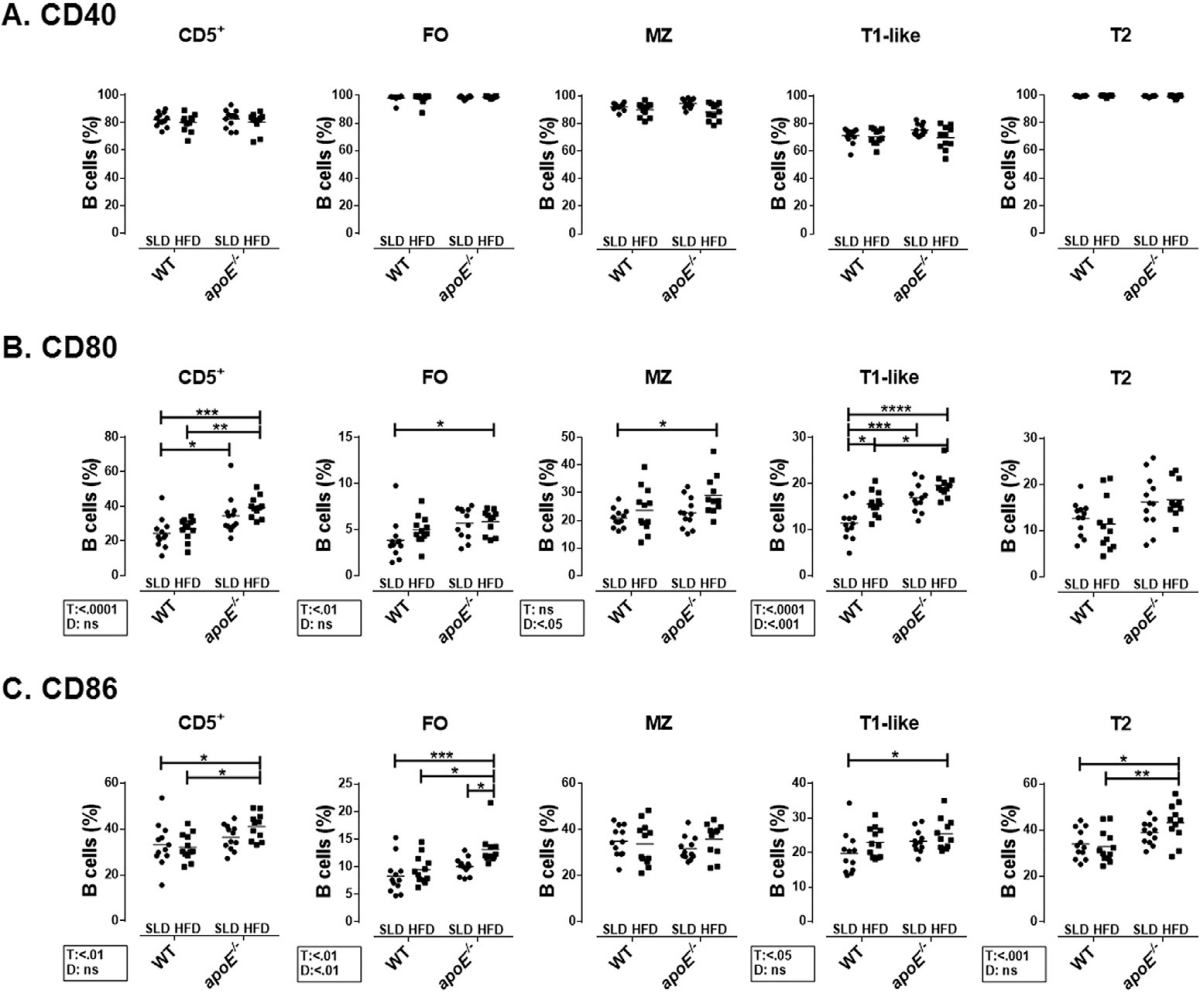
**Frequency of CD40**^**+**^**, CD80**^**+**^**and CD86**^**+**^**B cell subpopulations from mice with atherosclerosis**. Frequency of **A.** CD40^+^, **B.** CD80^+^ and **C.** CD86^+^ on splenic B cell subpopulations. Mean and data from 11-12 mice per group are shown from three independent experiments. Analysis was performed as in Figure 4. Two-way ANOVA with Šídák post-test were performed.

**Fig. 7:**
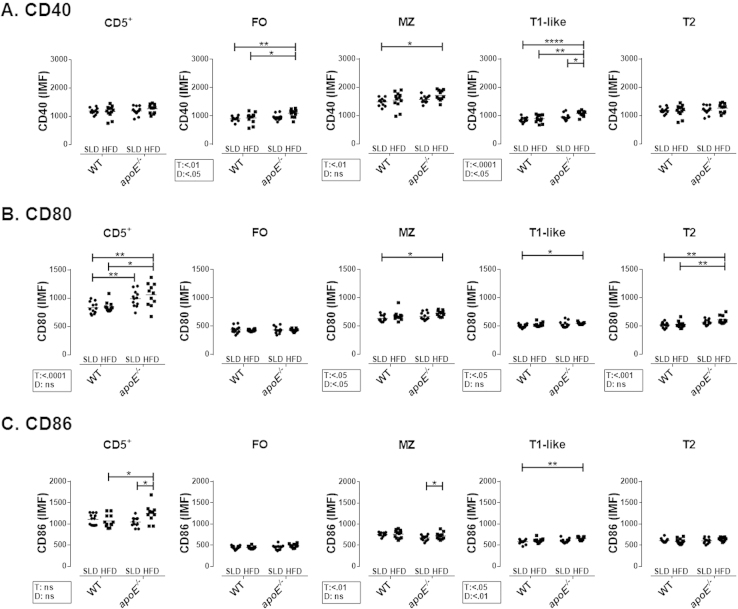
**Expression of CD40, CD80 and CD86 on B cell subpopulations from mice with atherosclerosis**. MFI of **A.** CD40, **B.** CD80 and **C.** CD86 on splenic B cell subpopulations. Mean and data from 11-12 mice per group are shown from three independent experiments. Analysis was performed as in Figure 4. Two-way ANOVA with Šídák post-test were performed.

**Fig. 8:**
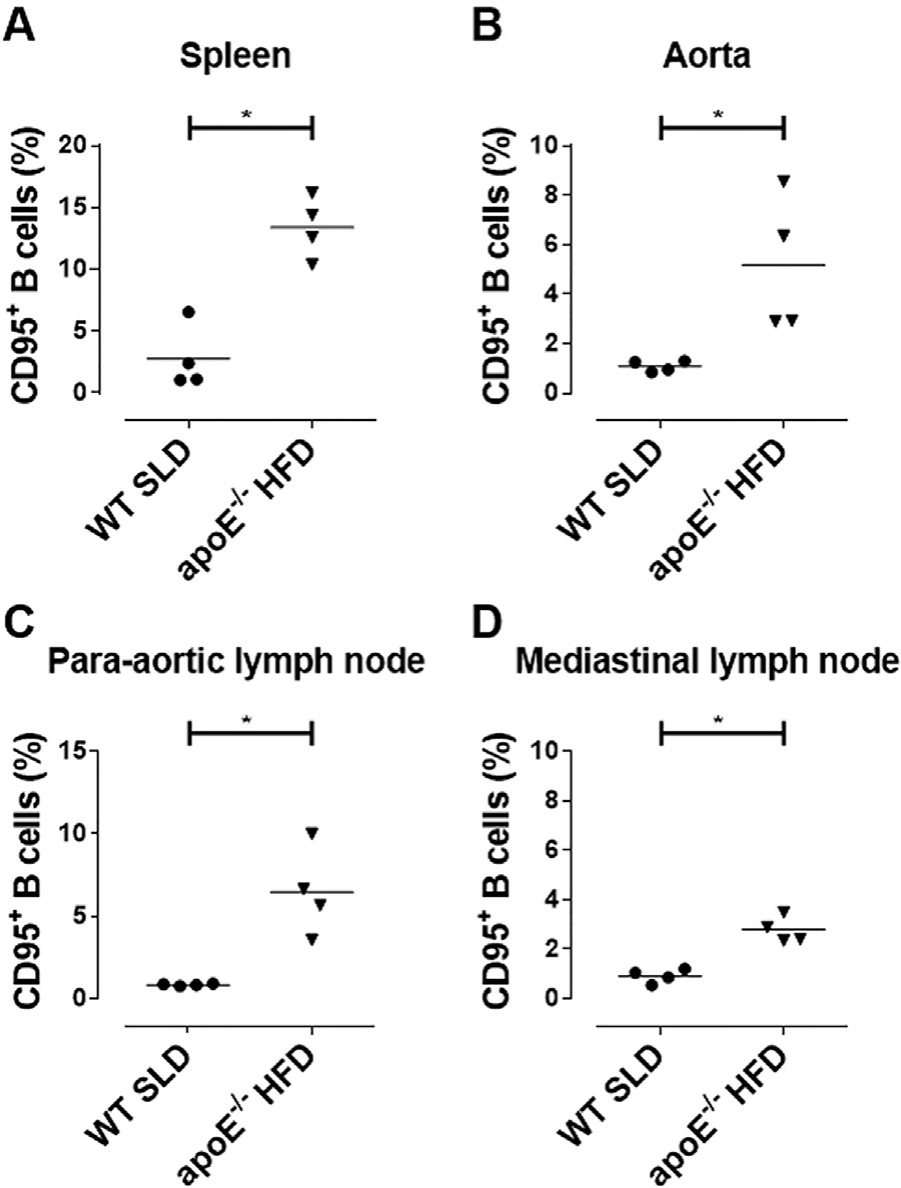
**Frequency of CD95**^**+**^**B cells in WT-SLD and*****apoE***^***-/-***^**HFD mice.** The percentage of CD95^+^ B cells from **A.** spleen, **B.** aorta, **C.** para-aortic and **D.** mediastinal lymph nodes. Mean and data from 4 mice per group are presented. Mann-Whitney test. * p<0.05.

**Fig. 9:**
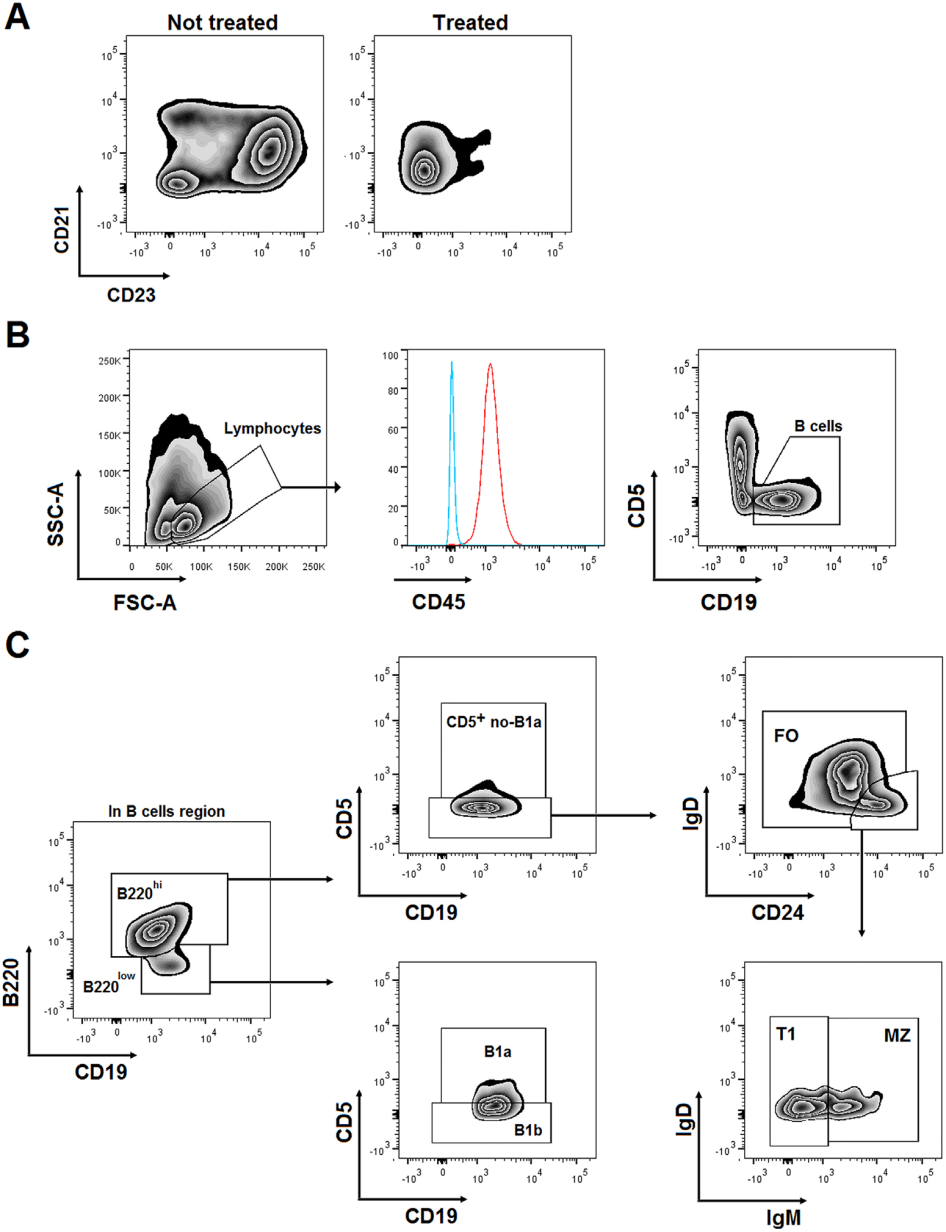
**Analysis of B cells of aortic tissue. A.** Representative density plot of CD21 and CD23 expression on splenic cells from a WT-SLD mouse with or without enzymatic cocktail treatment. **B.** Representative selection of aortic lymphocytes from a WT-SLD mouse according to the SSC-A and FSC-A parameters, with a typical CD45 expression of aortic lymphocyte cells (Red hystogram) and their analysis based on CD5 and CD19 expression. **C.** Representative density plots of the analysis of aortic B cell subpopulations in a WT-SLD mouse. According to the expression of CD19, B220, CD5, CD24, IgD and IgM, lymphocytes were divided into B1a, CD5^+^ non-B1a, B1b, FO, MZ and T1 subsets.

**Fig. 10:**
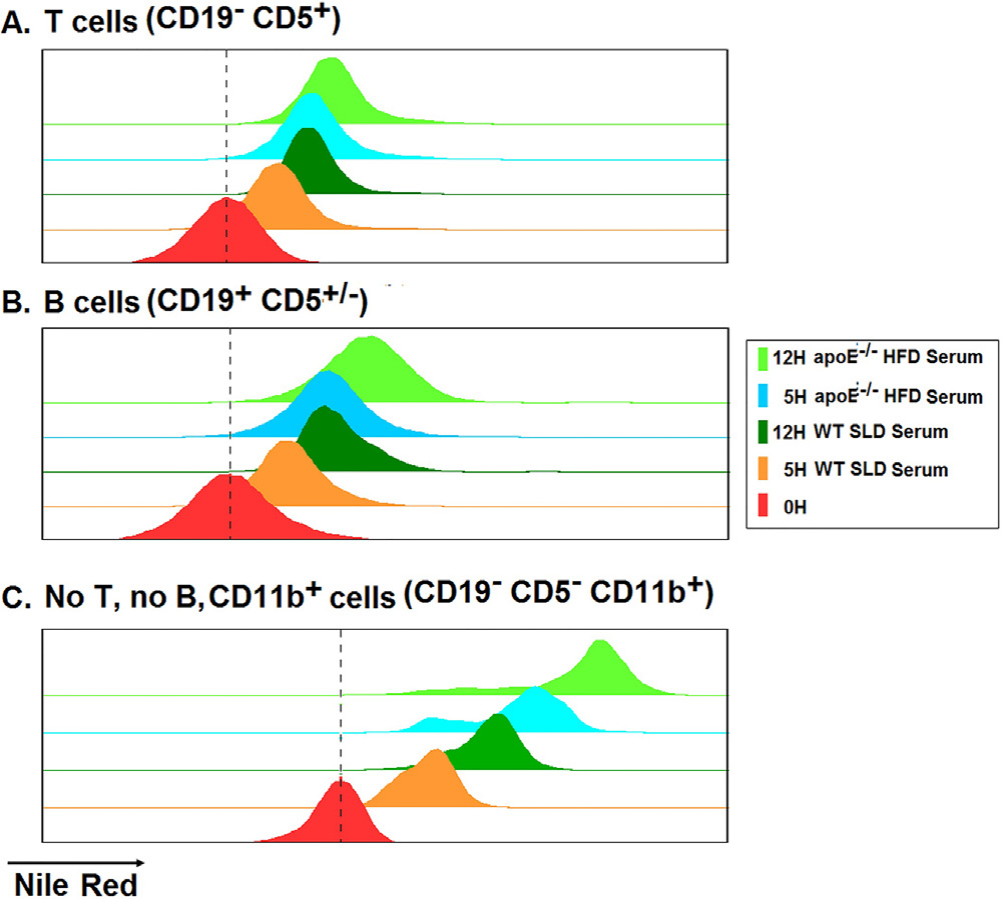
**Lipid accumulation in splenocytes exposed to lipemic serum.** Representative histograms of **A.** T cells (CD19^-^CD5^+^), **B.** B cells (CD19^+^) and **C.** CD11b^+^ myeloid cells (CD19^-^CD5^-^CD11b^+^) at 0, 5 and 12 h of culture of splenocytes from *apoE*^*-/-*^ mice of 8 weeks old with serum from *apoE*^*-/-*^ HFD or WT-SLD mice. The dotted line indicates the MFI of Nile Red at the beginning of the culture (0 h).

**Fig. 11:**
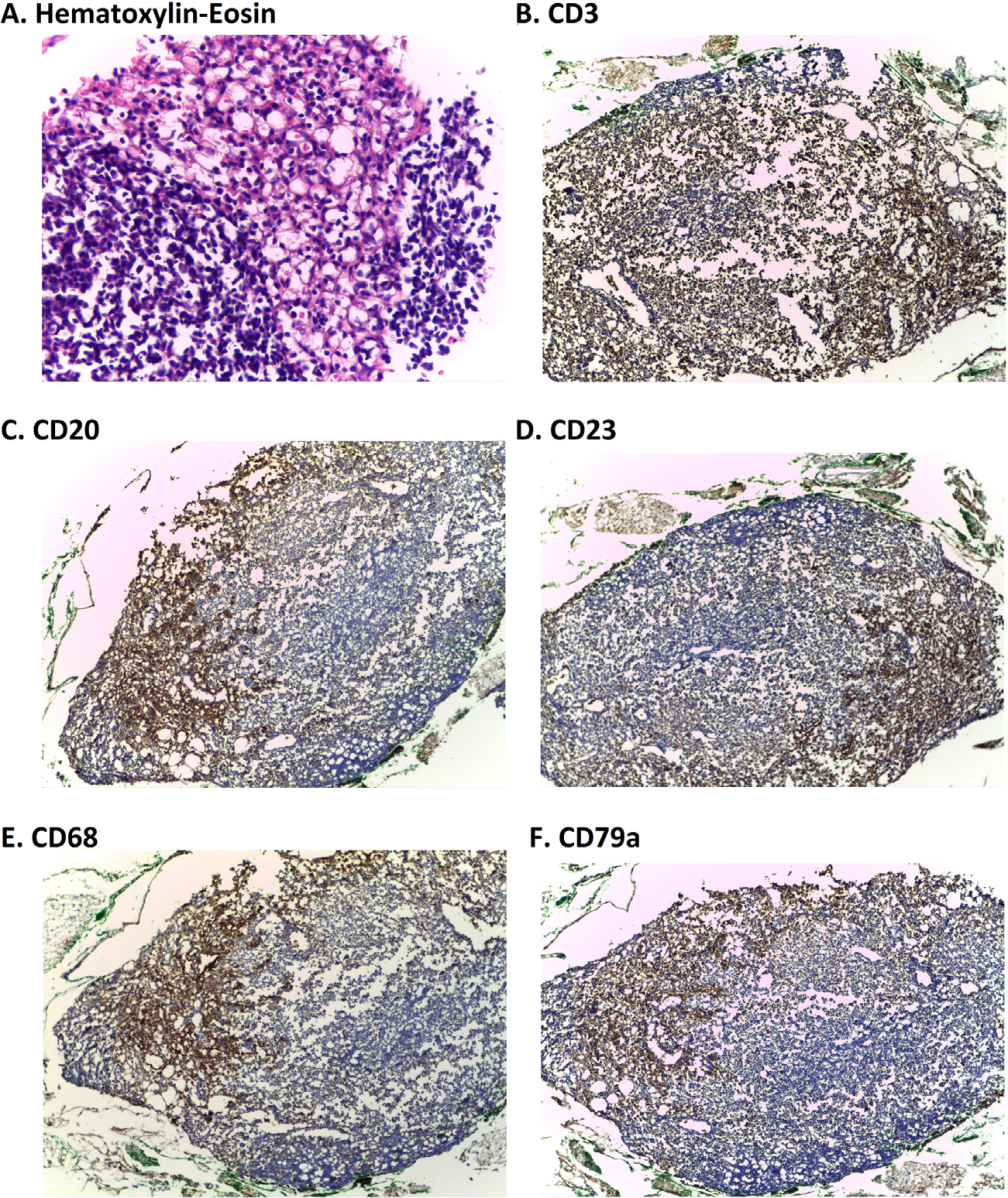
**Similar CD3, CD20, CD23, CD79a and CD68 Immunostaining among the four study groups in lymph nodes.** Representative pictures of the staining of para-aortic lymph nodes from the four study groups at 4X of magnification with **A.** Hematoxylin-Eosin, **B.** CD3, **C.** CD20, **D.** CD23, **E.** CD68 and **F.** CD79a.

**Fig. 12:**
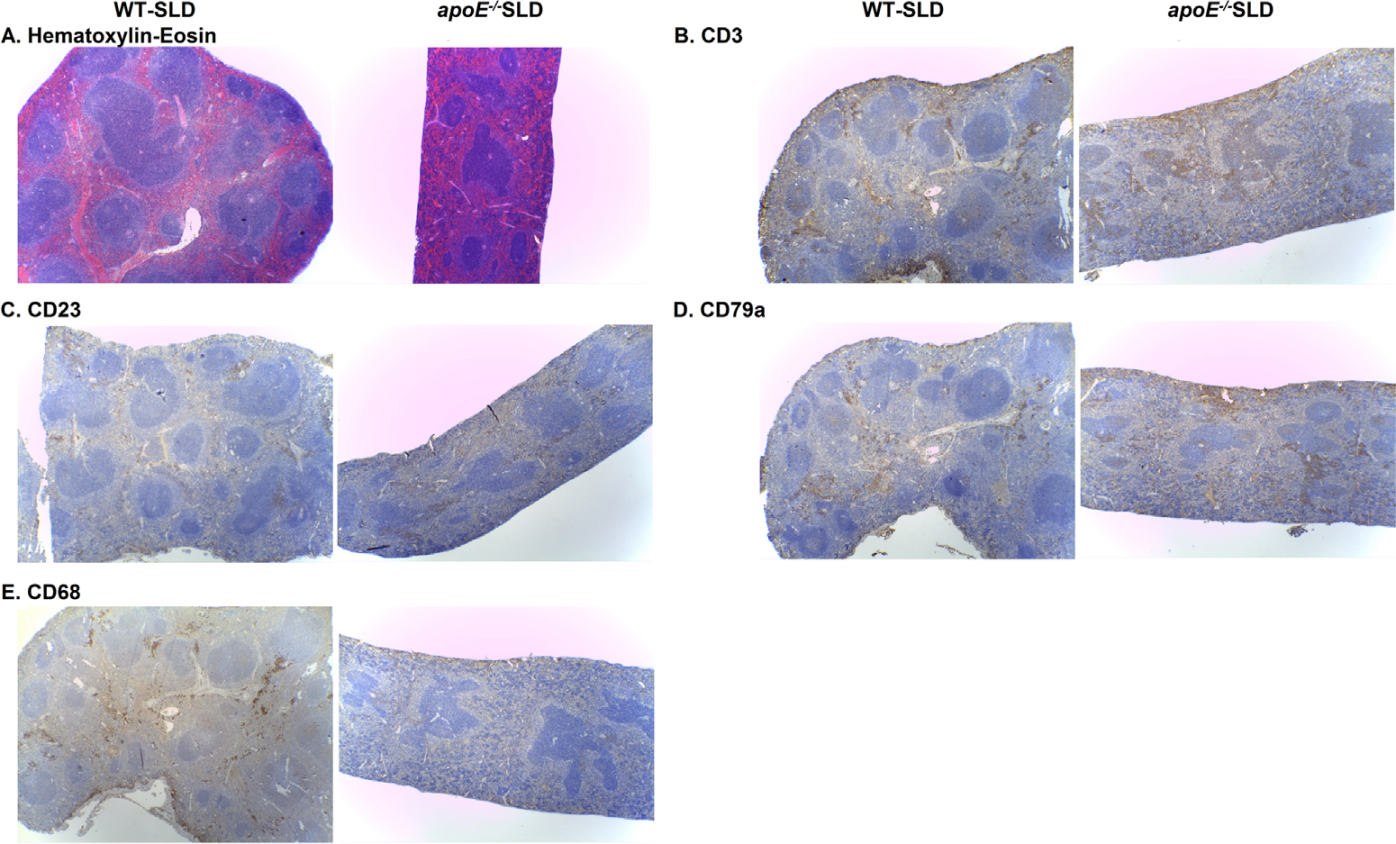
**Similar CD3, CD23, CD79a and CD68 Immunostaining between WT-SLD and*****apoE***^***-/-***^**SLD in spleens.** Representative photographs of the staining of spleen form WT-SLD (left) and *apoE*^*-/-*^SLD mice (right) at 4X of magnification with **A.** Hematoxylin-Eosin, **B.** CD3, **C.** CD23, **D.** CD79a and **E.** CD68.

**Fig. 13:**
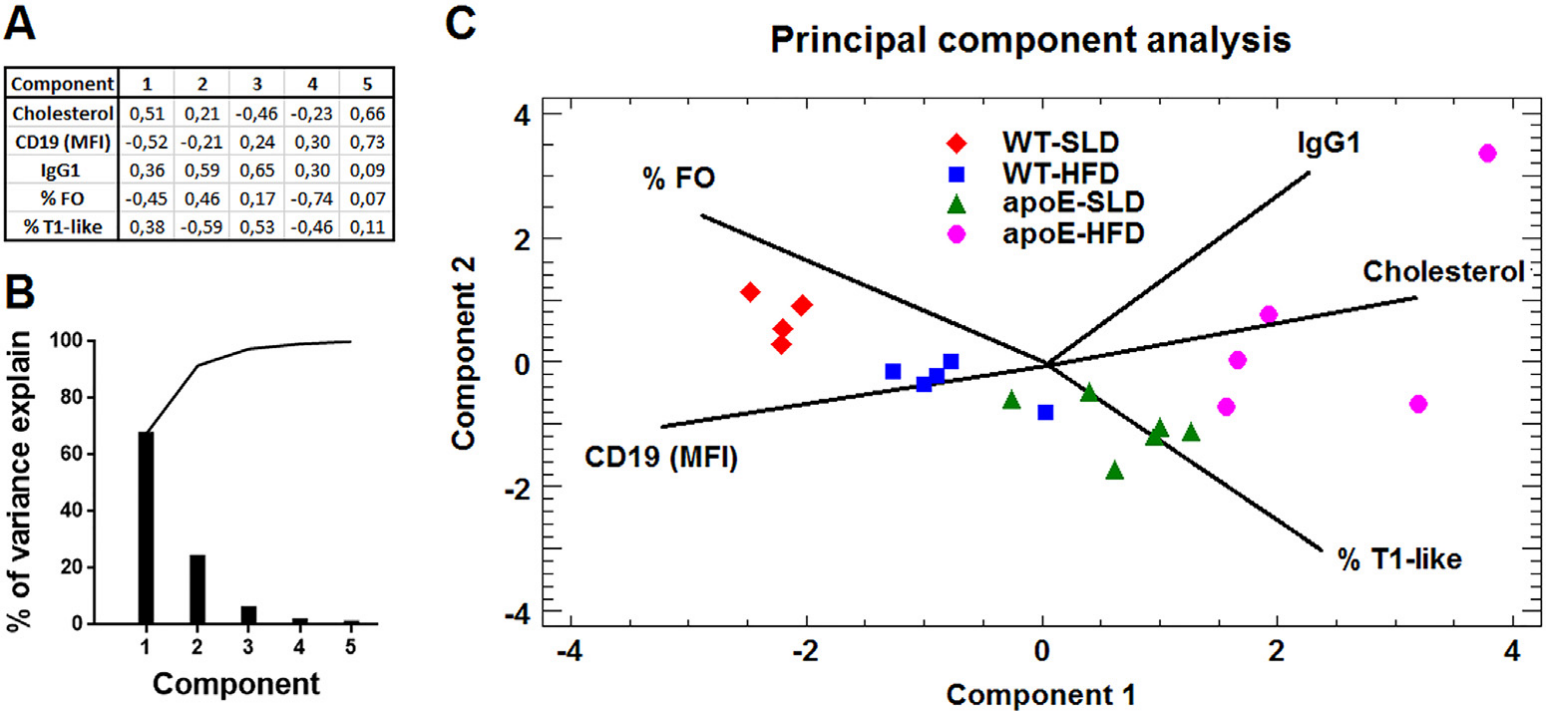
**Principal component analysis of alterations found in B cells. A.** Table of weights of each variable in the components. **B.** Pareto plot showing the contribution of each component to the overall variance in the analysis. **C.** Two-dimensional graph displaying the principal component analysis from 5-6 mice per group.

**Fig. 14:**
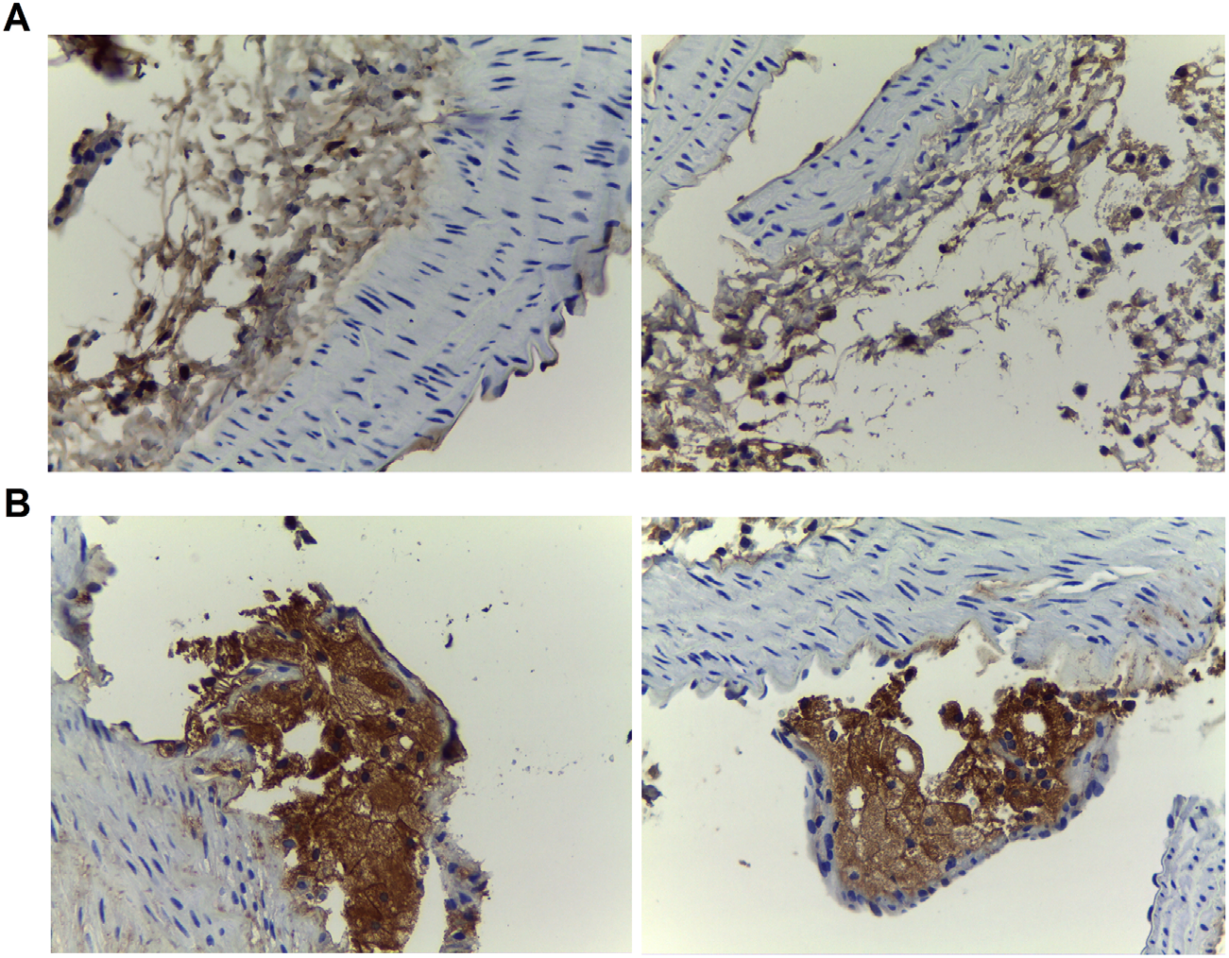
**Presence of B cells and macrophages in atherosclerotic plaque from aorta.** Representative photographs of the labeling of aortic tissue form *apoE*^*-/-*^SLD mice at 10X of magnification with **A.** CD20 and **B.** CD68 (brown).
